# Supplementary material for: Quality and Quantity of Protein Intake Influence Incidence of Type 2 Diabetes Mellitus in Coronary Heart Disease Patients: From the CORDIOPREV Study
Source: Nutrients. 2021 Apr 7;13(4):1217. doi: 10.3390/nu13041217 (PMC8067822; doi:10.3390/nu13041217)
Supplement: Supplementary file 1 [file nutrients-13-01217-s001.zip › nutrients-1142219-SI.odt]

Figure S1: Average daily food groups intake at baseline and after 1 year of intervention. Intake of food groups is expressed as g per 1000 kcal, and it is categorized by study groups. Between-group differences were assessed using Wilcoxon rank sum test for unpaired data and within-group differences were assessed using Wilcoxon signed rank test. *p<0.05 for comparisons between groups at year 1 of follow-up. # p < 0.05 for comparisons with baseline in each group.
